# Nano-cones for broadband light coupling to high index substrates

**DOI:** 10.1038/srep38682

**Published:** 2016-12-07

**Authors:** J. Buencuerpo, L. Torné, R. Álvaro, J. M. Llorens, M. L. Dotor, J. M. Ripalda

**Affiliations:** 1 IMM-Instituto de Microelectrónica de Madrid (CNM-CSIC), E-28760 Tres Cantos, Madrid, Spain

## Abstract

The moth-eye structure has been proposed several times as an antireflective coating to replace the standard optical thin films. Here, we experimentally demonstrate the feasibility of a dielectric moth-eye structure as an antireflective coating for high-index substrates, like GaAs. The fabricated photonic crystal has Si_3_N_4_ cones in a square lattice, sitting on top of a TiO_2_ index matching layer. This structure attains 1.4% of reflectance power losses in the operation spectral range of GaAs solar cells (440–870 nm), a 12.5% relative reduction of reflection power losses in comparison with a standard bilayer. The work presented here considers a fabrication process based on laser interference lithography and dry etching, which are compatible with solar cell devices. The experimental results are consistent with scattering matrix simulations of the fabricated structures. In a broader spectral range (400–1800 nm), the simulation estimates that the nanostructure also significantly outperforms the standard bilayer coating (3.1% vs. 4.5% reflection losses), a result of interest for multijunction tandem solar cells.

Optical losses (reflection and parasitic absorption) in solar cells are typically minimized by using thin film dielectric multilayer coatings with high transmittance^1^,^2^. These designs are limited to combinations of different materials and thicknesses. Nanostructured antireflective coatings have been proposed several times in the literature as a promising alternative^3^,^4^,^5^,^6^,^7^,^8^,^9^,^10^,^11^,^12^,^13^,^14^,^15^,^16^,^17^,^18^,^19^,^20^, but their full potential is yet to be achieved. This approach presents the opportunity to engineer the optical response from the top layer by modifying the in plane structures. The bio-mimetic moth-eye nanostructure typically presents low reflectance as previous research has documented^6^,^7^,^9^,^11^,^12^,^13^,^14^,^15^. However, the previous studies did not consider the case of transparent dielectric nanostructures on high index substrates such as Si, GaAs or GaInP. In particular, in refs 9, 13 and 15 the nanostructure is sits on top of a relatively low index substrate, relaxing the requirements of the anti-reflective coating. A key limitation of these proposals is that they cannot be directly applied to inorganic solar cells given the high refractive index of the semiconductor. On the other hand, the nanostructures in refs 6, 7, 11 and 14 sit on top of high index substrates, but the semiconductor material itself is nanostructured rather than a transparent dielectric. The major drawback of nanopatterning the semiconductor is increasing surface recombination^21^, so an electrical degradation of the device is expected and a better passivation is needed. In conclusion, nanostructuring laterally some of the layers is increasingly seen as a requirement for optimal solar cell efficiency, as evidenced by recent literature^3^,^4^,^5^,^6^,^7^,^8^,^9^,^10^,^11^,^12^,^13^,^14^,^15^,^16^,^17^,^18^,^19^,^20^.

Not every nano-lithography technique is suitable for low cost fabrication of large area devices such as solar cells. We have chosen laser interference lithography (LIL) for its capability to uniformly pattern large areas (>1 m^2^)^22^,^23^. Electron beam lithography (EBL) and focused ion beam (FIB) are often used for fabricating high quality photonic crystals, but these techniques are not suitable for large area applications due to the use of finely focused beams to define the patterns point by point. Besides its large area capability, another considerable advantage of LIL is that it is a mask-less technique, in contrast with nano-imprint lithography, and therefore, it is compatible with preexisting patterns such as solar cell electrical contacts. Furthermore it does not present the problems associated with template degradation by particle contamination that are often associated with nano-imprint and contact photolithography.

In this work, we have fabricated a moth-Eye nanostructure made with a transparent dielectric (Si_3_N_4_) on a high index substrate, GaAs. The design was globally optimized using 3D simulations in a previous work^24^. The design consists of Si_3_N_4_ nano-cones in a square lattice with the following optimal dimensions: period *a* = 344 nm, height *H* = 512 nm, and radius *R* = 163 nm. Between the nanostructure and the substrate there are two index matching thin films, one made of Si_3_N_4_ (*d* = 49 nm), like the nanostructure, and one of TiO_2_ (*d*_0_ = 51 nm). These dimensions were used as a target for fabrication.

## Experimental methods

Our structure is fabricated on a GaAs substrate and optimized for III–V tandem solar cells. These cells generally operate in optical concentration conditions, allowing to reduce the semiconductor area by up to three orders of magnitude. Still, the fabrication techniques used in this paper are fully compatible with large area solar cell production. The TiO_2_ deposition is done using atomic layer deposition (ALD) and the Si_3_N_4_, using plasma enhanced deposition (PECVD). Both techniques are extensively used in electronics and photovoltaics^25^,^26^,^27^,^28^,^29^,^30^. On the other hand, the nanostructure is patterned by LIL and it is transferred to silicon nitride by reactive ion etching (RIE). LIL is suitable for photovoltaics as discussed in the introduction, whereas RIE is widely used in photovoltaics and microelectronics^31^,^32^,^33^,^34^,^35^.

The initial step for the fabrication was the deposition of 51 nm of TiO_2_ using ALD. Subsequently 616 nm of Si_3_N_4_ were deposited using PECVD. Both layers were deposited at a relatively low temperature (200 °C) to ensure process compatibility with a range of photovoltaic technologies. The samples were covered with a diluted positive photoresist (S1805 + PGMEA 1:1) by spin coating at 5000 rpm for 60 seconds and baked at 115 °C for one minute before exposure. The obtained photoresist thickness was 150 nm. We did not use a bottom antireflective resist, as the sample itself is a better ARC.

An auxiliary pattern consisting of an array of 50 *μ*m wide lines with a period of 500 *μ*m was defined by exposing the photoresist using conventional proximity mask UV lithography. This pattern was used to measure the height after the etching step using a profilometer. The sample was then exposed using LIL with a Lloyd mirror configuration and a 12 mW, 405 nm semiconductor diode laser on a temperature controlled mount. The light from the laser was spatially filtered using a lens (*f* = 4 mm) and a pin hole (5 *μ*m) at the focal plane. A distance of 35 cm separated the pin hole from the sample and the aluminum mirror. The exposure intensity over the sample was controlled using a GaP photodiode mounted below the sample mount.

**Figure 1:**
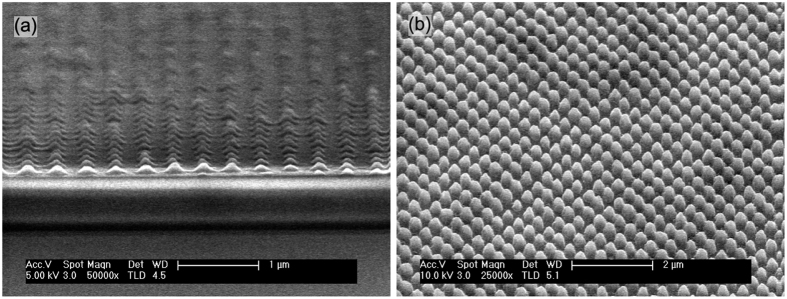
(**a**) SEM image of the photoresist nanopatterned before the RIE etching step. (**b**) SEM image of the nanostructure after the RIE etching.

To obtain a square lattice (2D) the pattern must be exposed twice in two orthogonal directions. The pattern inside the unit cell will be similar to a square or a circle depending one the exposure dose^36^. We have aimed at fabricating a circular nanostructure, so the exposure dose was near to the saturation of the photoresist. After the exposure, the sample was immersed in the developer 15 seconds to obtain the pattern. Figure 1(a) presents the sample after the lithography. The thickness of the photoresist was reduced by the developing process (100 nm) since the nanostructure size is below the resolution specified by the manufacturer of the photoresist and the stability of the photoresist is compromised for patterns smaller than 500 nm. This limits the final nanostructure height, as the photoresist was used as mask for the etching step.

The pattern was transferred to the Si_3_N_4_ layer using RIE with a 200 W, 460 V, 10 mTorr plasma with a gas flow of 25 sccm of N_2_ and 25 sccm of CHF_3_^37^. The attack rates obtained were 55–60 nm/min for the Si_3_N_4_ and 15–20 nm/min for the photoresist. In order to avoid excessive thermal damage to the photoresist, the attack was pulsed following a periodic sequence of 30 seconds of plasma, 2 minutes of vacuum, 1 minute of N_2_ flux and 1 minute of vacuum. To complete the dry etching the loop was repeated 20 times, namely 10 minutes of plasma. The final height of the nanostructure was measured using a profilometer, obtaining 390 ± 10 nm. Figure 1(b) shows the sample after the etching process. After the dry etching, the sample was cleaned using a 50 WO_2_ plasma during 30 seconds to remove possible photoresist residues.

The reflectance was measured using the light from a halogen lamp dispersed by a 0.3 m focal length monochromator. Temporal fluctuations of the excitation beam were compensated with a reference silicon photodiode. The beam was modulated at 477 Hz with a chopper to allow for lock-in signal detection. A 200 micron core size multi-mode optical fiber was used to allow for mechanical decoupling of the monochromator and the rest of the set up. The collimated excitation beam was aligned with a Glan-Thompson polarizer at one the horizontal arms of a 45 degree beam splitter, and then focused to a 30 *μ*m diameter spot using a 20X magnification objective lens (NA = 0.4) at the lower vertical arm of the beam splitter. The signal reflected from the sample was collected by the objective and sent through the beam splitter to a lens focused on a silicon photodiode on the upper arm of the beam splitter. For measurements at wavelengths above 700 nm, a dichroic filter was used to block short wavelength light due to the second diffraction order from the monochromator. For our experimental set up we choose optical elements optimized for visible and near infrared transmission. The bounds of our experimental spectra (440 nm to 870 nm) are also limited by the position of the GaAs absorption edge and the spectral distribution of our illumination source.

## Results and Discussion

**Figure 2:**
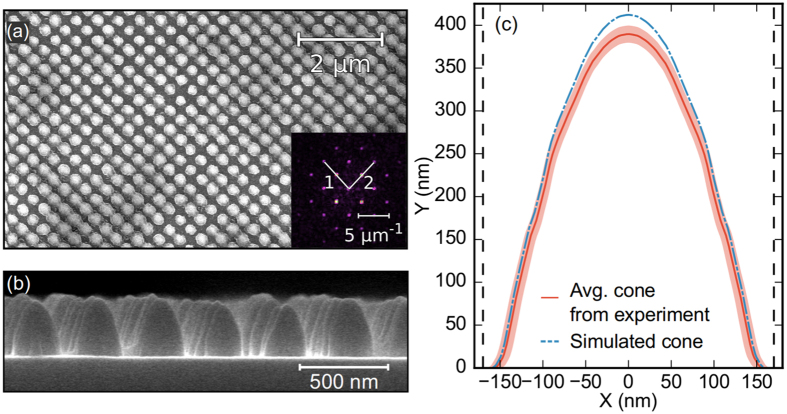
(**a**) SEM image of the nanostructure, inset fast Fourier transform (FFT) of the structure where 1 and 2 (white-lines) are the (2, 0) and (0, 2) lattice vectors, whose moduli correspond to a periodicity of 339 ± 3 nm. (**b**) Cross sectional SEM image used to estimate the mean profile. (**c**) Mean profile of the cones, scaled to the experimental data (red line), and optimal profile extracted from the reflectance fitting (blue dash-dotted line). The optimal lattice parameter is *a* = 340 nm (vertical dashed black).

The reflectivity measurements were compared with the simulations results obtained using the scattering matrix method (SMM)^38^. The refractive indices of the materials were taken from refs 39, 40, 41, 42. The most rigorous comparison between experiment and simulation would require the simulation of a super-cell of the measured nanostructure (a spot of ~30 *μ*m radius), as the fabricated nanostructure is not perfectly homogeneous, see Figs 1(b) and 2(b). Unfortunately, this would require huge computational resources. Alternatively, an average unit cell can quantitatively describe the reflectance of the system. The dimensions describing this unit cell should be representative of the distribution of the real nanostructures. An initial set of dimensions describing the size and shape of the nanostructures was obtained from electron microscopy images and profilometer measurements and then slightly refined by fitting the simulations to the experimental reflectivity. The lattice parameter, *a*, was extracted from Fig. 2(a). We have used Fig. 2(b) to estimate an average profile of the fabricated cone. This average profile was smoothed using a Savitzky-Golay filter assuming that the average profile has rotational symmetry around a vertical axis, see Fig. 2(c). The cone is approximated in the SMM code as a stack of 50 concentric cylinders.

**Figure 3:**
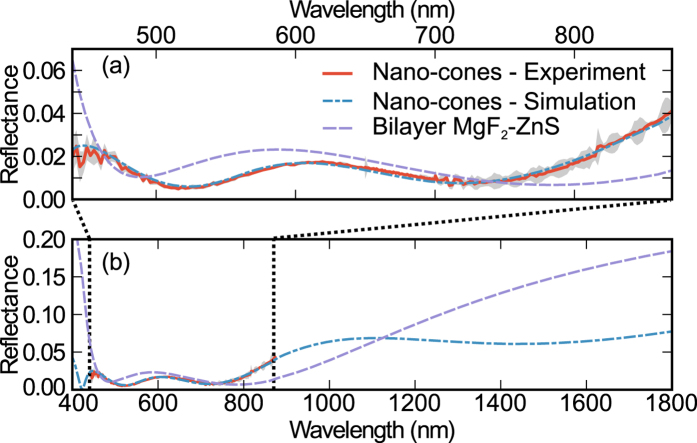
(**a**) Experimental reflectance (red), experimental standard deviation (light gray), theoretical reflectance (dashed-dotted blue) and theoretical reflectance of an optimal bilayer (MgF_2_-ZnS) (dashed purple). (**b**) Theoretical reflectance in an extended spectral range.

The experimental and simulated reflectance data are shown in Fig. 3(a). Table 1 compares the nanostructure dimensions obtained from sample topography measurements with those obtained from fitting the simulations to the reflectance data. The fitting accurately reproduces the experimental results without large deviations in the fit parameters from their expected values. The experimental thickness for the TiO_2_, *d*_0_, is 5 nm bigger than the fitted one. This small difference is most likely a result of the GaAs native oxide not being included in our simulations. An important question might arise in view of the SEM images presented in Figs 1 and 2: the role of the inhomogeneities introduced during fabrication. Our structures show an excellent uniformity in the lattice period, denoted by the sharp peaks in the Fourier transform shown in the inset of Fig. 2(a). We have also achieved a high uniformity in the height of the nanostructures, as can be inferred after inspecting Figs 1(b) and 2(b). The only parameter showing some non-uniformity is the filling factor, i.e. the base radius of the cone. Many studies have been performed around the role of the disorder in the performance of a nanostructured light-trapping structure. These studies are mostly focussed on the impact of disorder in the lattice period, while disorder in the pattern size generally plays a secondary role. Indeed, as Burresi *et al*. indicated in ref. 43, the low order Mie resonances of the nanostructures are spectrally very broad and these resonances are the most relevant ones in this context, given that this antireflective coating operates in the subwavelength regime. In terms of light scattering, this means when the wavelength is larger than the size of the scatterer. A few studies support this operating principle. It has been shown that in disordered 1D structures, the reflection losses decrease with the degree of disorder of the groove width at low degree of disorder of the period^44^. Similar results have been reported for 2D structures^45^,^46^, like the one studied here. Hence, the non-uniformity in the filling factor does not necessarily introduce a penalty in the performance of the AR, on the contrary, it might be beneficial to reduce the reflection losses. From our numerical analysis of the reflection data, we conclude that the local changes in the filling factor do not have a noticeable impact in the global performance of the structure.

Figure 3(b) shows the reflectance resulting from our fitting in an extended spectral range from 400 to 1800 nm in comparison with the optimized bilayer. To estimate the total power loss due to reflection we have integrated the reflectance weighted with the solar spectral irradiance:


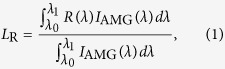


being *I*_AMG_(*λ*) the spectral irradiance, and *R(λ*) the reflectance.

In the visible and near infrared range of the spectrum (*λ*_0_ = 440 nm and *λ*_1_ = 870 nm), the nanostructures lead to a 12.5% relative reduction of reflection power losses in comparison with a standard bilayer (1.4% vs. 1.6% absolute power loss). Extending the integration range to *λ*_0_ = 400 nm and *λ*_1_ = 1800 nm, our simulations predict a reflection power loss of 3.1%, while the bilayer leads to a 4.5% power loss. Thus, a 31% relative reduction of power losses is expected. As seen in Fig. 3(b), the nano-cones significantly outperform the bilayer at the shortest wavelengths. However, as the light wavelength shortens the reflectance is expected to become sensitive to the exact shape and size of the nanostructures, thus extending the integration range to shorter wavelengths would have introduced larger uncertainties in the obtained power losses. On the other hand, for long wavelengths, the nanostructure is averaged, and we are able to estimate the results for a broader spectrum. For long wavelengths, the nanostructure has lower reflectance from 1120 to 1800 nm.

The absorption losses can be estimated by replacing the reflectivity with the absorption of the ARC layers in Eq. (1) 24. For the wider spectral range (400–1800 nm) the absorption loss of the nanostructured and the bilayer ARC are 0.09% and 0.29%, respectively. In both cases absorption only occurs for wavelengths shorter than 440 nm and in the higher index layers: TiO_2_ (nano-cones ARC) and ZnS (bilayer). The higher absorption in the bilayer results from the higher extinction coefficient of ZnS vs. TiO_2_. In fact, the measured extinction coefficient of our TiO_2_ layers (see Supplementary Material), discards the possibility of significant absorption losses in the ARC.

This design has been proved to be superior compared to a standard bilayer, but it is important to put it in the context of state of the art designs presented in the literature. Perl *et al*. reported in ref. 19 a theoretical 1% power loss integrated over the whole solar spectrum on a GaInP substrate and a 2.8% experimental reflectance loss on a 1 *μ*m thick GaInP layer on a GaAs substrate. This design is based on a SiO_2_ cone-like nanostructure on top of a stack of 7 SiO_2_ and TiO_2_ layers. To enable a direct comparison, we have replaced the GaAs substrate in our simulations of the fabricated structure with the GaInP and GaInP/GaAs substrates used in ref. 19, as the higher refractive index of GaAs leads to higher optical power losses. The reflectance power loss of our optimal structure on a GaInP substrate is 0.9%, and 1% on GaInP/GaAs^24^. The nanostructures resulting from our fabrication efforts are shorter, narrower, and less sharp than the optimal ones resulting from our simulations. Simulating our fabricated nanostructures on top of the substrates used by Perl *et al*., the resulting reflection losses are 2.4% on GaInP and 2.5% on GaInP/GaAs. Based on these results it can be concluded that our design results in similar reflectance losses than those of the hybrid design proposed by Perl *et al*. The main difference is that the here proposed structure requires a single TiO_2_ layer rather than a SiO_2_/TiO_2_ multilayer under the nanostructures. Our previously published analysis suggests that photonic effects such as coupling to quasi-guided modes are fundamental to achieving a very low reflectance with our relatively simple design^24^. Such effects are not expected in the case of the structure proposed by Perl *et al*. due to the very low refractive index contrast of SiO_2_ with air. In our case, the use of fully 3D simulations in the optimization of the nanostructures allowed for the tuning of photonic effects beyond the simple graded effective index effect that has been studied in previous efforts based on 1D simulations.

### Reflectance vs. the incident angle

**Figure 4:**
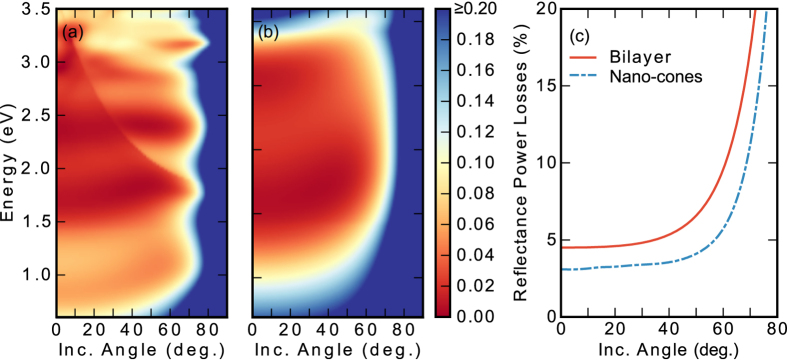
Theoretical reflectance as a function of the incident angle of the cone nanostructure (**a**) and for the optimized bilayer (**b**). (**c**) Reflectance power losses as a function of the incident angle of the cone nanostructure (blue dash-dotted) and the optimized bilayer (red line).

The nano-cone exhibits lower reflectance losses than the standard bilayer at normal incidence. Still, concentrator optics leads to an incident angle distribution^47^, therefore it is important to consider the reflectance power losses for angles out of the normal incidence. The reflectance as a function of the incident angle is illustrated in Fig. 4(a) and (b), for the nanostructure and the bilayer respectively, whereas the integrated power losses for both cases are shown in Fig. 4(c). As seen in Fig. 4(a), the low reflectance of the nanostructure is kept for oblique incident angles. In fact, the reflectance power losses for the nanostructure is below 5% from 0 to 57 degrees of incident angle, whereas the standard coating reach 5% power losses from 34 degrees and above, see Fig. 4(c). These results for the fabricated cone structure are very similar to those found for the ideal cone structure^24^, which supports the robustness of the design.

## Conclusions

In conclusion, our results indicate that it is possible to significantly decrease the reflectance losses on high index substrates using an optimized moth-eye photonic crystal. In fact, the nano antireflective coating has proved to obtain lower reflectance losses than a standard coating. The fabricated nanostructured antireflective coating represents a 31% relative reduction of reflection power losses over the standard bilayer ARCs. Still, ample room for improvement exists by further optimizing the here proposed fabrication process. In particular, a shorter wavelength laser and a higher resolution photoresist might improve the lithography. A thicker mask for the RIE dry etching step would lead to taller nanostructures. Our simulations predict that further progress along this line of research should lead to solar cells with optical losses under 1% using the here proposed antireflective nanostructures.

## Additional Information

**How to cite this article**: Buencuerpo, J. *et al*. Nano-cones for broadband light coupling to high index substrates. *Sci. Rep.* **6**, 38682; doi: 10.1038/srep38682 (2016).

**Publisher's note:** Springer Nature remains neutral with regard to jurisdictional claims in published maps and institutional affiliations.

## Supplementary Material

Supplementary Materials

## Figures and Tables

**Table 1:** Nanostructure dimensions obtained from sample topography measurement (“Experiment”), and dimensions obtained from fitting the simulations to the reflectance data (“Simulation”).

System	*a* (nm)	*R* (nm)	*H* (nm)	*d* (nm)	*d*_0_ (nm)
Experiment	336–342^(1)^	148–165^(2)^	380–400^(3)^	16–66^(4)^	51^(5)^
Simulation	340	159	413	15	45

*a*^(1)^ and *R*^(2)^ are obtained from Fig. 2(a). (The radius distribution is presented in the supplementary materials); *H*^(3)^ is obtained from profilometer measurements, *d*^(4)^ is obtained from the etch ratios from the RIE, and 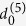 is obtained from the deposition by ALD.
