# Supplementary material for: Nano-cones for broadband light coupling to high index substrates
Source: Sci Rep. 2016 Dec 7;6:38682. doi: 10.1038/srep38682 (PMC5141501; doi:10.1038/srep38682)
Supplement: Supplementary Materials [file srep38682-s1.pdf]

Supplementary Materials: Nano-cones for broadband light coupling to high index substrates

J. Buencuerpo[1,*], L. Torne , R. Álvaro , J. M. Llorens , M. L. Dotor , and J. M. Ripalda

[1]IMM-Instituto de Microelectrónica de Madrid (CNM-CSIC), E-28760 Tres Cantos, Madrid, Spain
[*]j.buencuerpo@csic.es

Analysis of the filling factor inhomogeneities

The SEM image from Fig.2(a) from the main manuscript is digitally masked, and it is used to obtain the average radius. In Fig. S1 the mask and the histogram of the spatial distribution is presented. The radius obtained is R=156.5 $\pm$ 8.4 nm, after analyzing 494 cones.

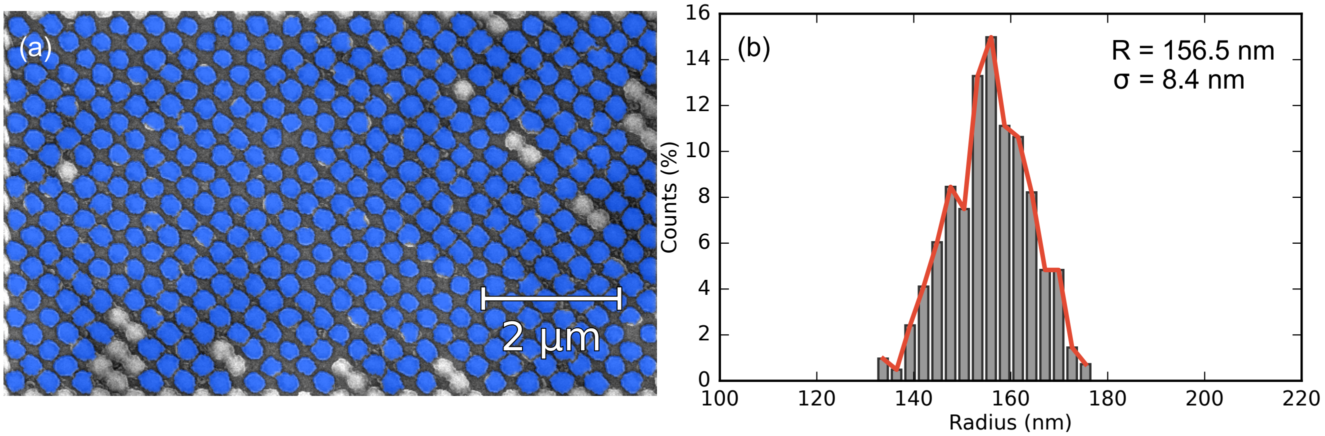

Figure S1. (a) Masked SEM image (blue) to determine the cones radius distribution. (b) Histogram of the radius from panel (a), (494 Counts).

Absorption losses in the ARC layer

The parasitic absorption inside the ARC can minimize the final transmission to the device, and consequently transparent materials should be used. In nature there are several dielectrics completely transparent in the useful part of solar spectrum range (400-1800 nm) as SiO_2, Si_3N_4 or MgF_2. Yet, these dielectrics have low refractive index, typically n$\lesssim$ 2. To avoid reflection losses a higher refractive index is needed to couple light from air to the semiconductor which has n$\gtrsim$ 3.5. Typically, the materials used are ZnS or TiO_2 with n$\simeq$ 2.4. However, these materials are not fully transparent, in other words, the extinction coefficient, α, is not zero in the whole spectral range. In Fig.S2 the α for ZnS and TiO_2 is presented.

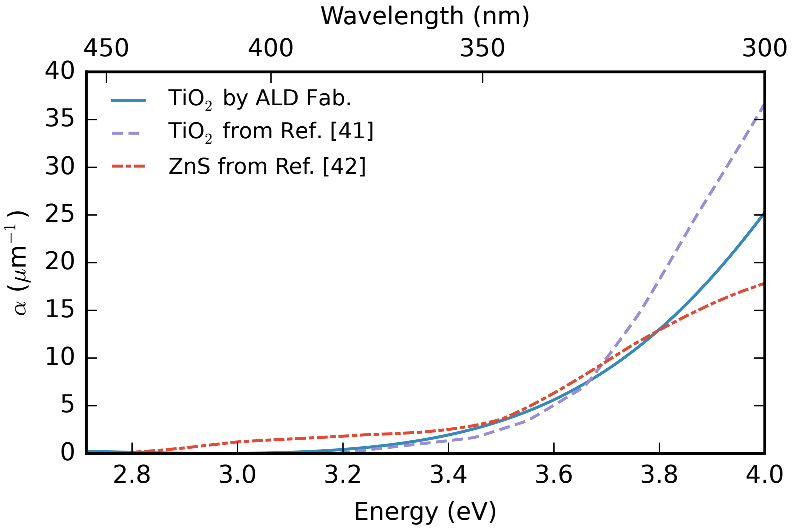

Figure S2. Extinction coefficient, α for the TiO_2 fabricated in this work (blue solid line), TiO_2 by Ref.[41] (purple dashed) and ZnS by Ref.[42]. (red dot dashed).

The parasitic absorption in the ARC TiO_2 layer appears for wavelengths shorter than 400 nm (3.1 eV), see Fig.S2. At this wavelengths the terrestrial solar spectrum decays sharply, therefore minimizing the power losses because of the absorption. Also the thickness of this layer is only 51 nm, leaving with residual reflection losses.

Sample Figures

In this section the sample is presented in different areas and scales, Fig. S3 and Fig.S4.

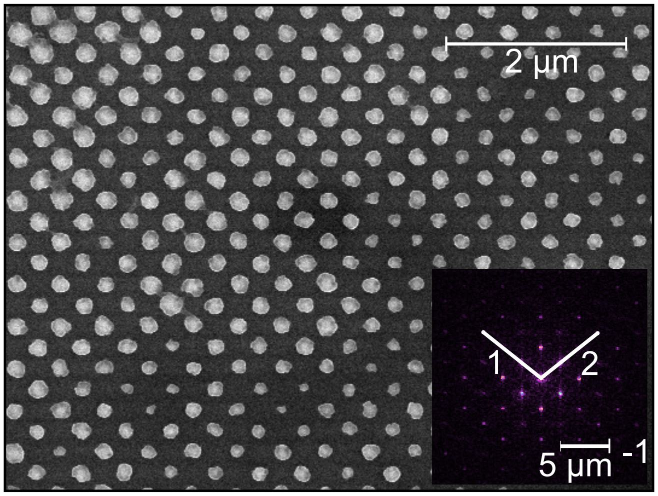

Figure S3. SEM image of the nanostructure, inset Fast Fourier transform (FFT) of the structure where 1 and 2 (white-lines) are the (3,0) and (0,3) lattice vectors, whose moduli correspond to a periodicity of 337 and 341 nm, respectively. This image shows the inhomogeneities appearing to the region near to the borders UV lithography, due to the over-exposition. This auxiliary pattern consists of an array of 50 μm wide lines with a period of 500 μm and it was defined to measure the height after the etching step using a profilometer. An image of the final sample is shown if Fig.S4(a). In an actual device this auxiliary pattern is unnecessary.

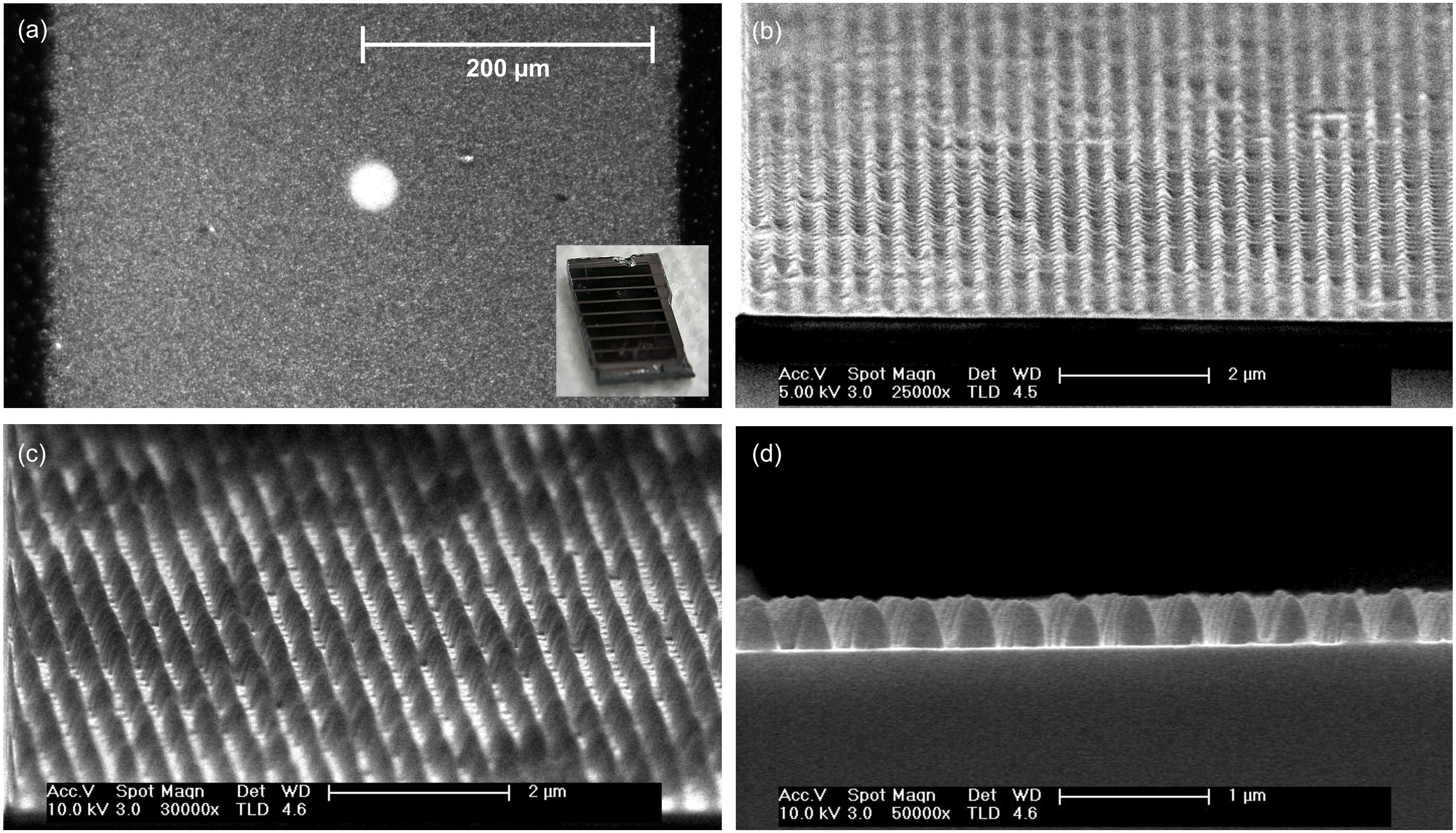

Figure S4. (a)Optical microscopy view of the sample under lateral and oblique illumination. The bright spot is the normally illuminated area for the reflectivity measurements. Inset, macroscopic view of half of the final sample. (b) SEM tilt image of the lithography before the etching. (c) SEM tilt image of the final nanostructures. (d) Cross-sectional SEM image of the sample.
